# The crosstalk within the breast tumor microenvironment in type II diabetes: Implications for cancer disparities

**DOI:** 10.3389/fendo.2022.1044670

**Published:** 2022-12-01

**Authors:** Christina S. Ennis, Pablo Llevenes, Yuhan Qiu, Ruben Dries, Gerald V. Denis

**Affiliations:** ^1^ Boston University-Boston Medical Center Cancer Center, Boston University School of Medicine, Boston, MA, United States; ^2^ Section of Hematology and Medical Oncology, Boston University School of Medicine, Boston, MA, United States; ^3^ Department of Pharmacology and Experimental Therapeutics, Boston University School of Medicine, Boston, MA, United States; ^4^ Division of Computational Biomedicine, Boston University School of Medicine, Boston, MA, United States; ^5^ Shipley Prostate Cancer Research Professor, Boston University School of Medicine, Boston, MA, United States

**Keywords:** type II diabetes mellitus, intercellular communication, tumor microenvironment, metabolic reprogramming, exosomes

## Abstract

Obesity-driven (type 2) diabetes (T2D), the most common metabolic disorder, both increases the incidence of all molecular subtypes of breast cancer and decreases survival in postmenopausal women. Despite this clear link, T2D and the associated dysfunction of diverse tissues is often not considered during the standard of care practices in oncology and, moreover, is treated as exclusion criteria for many emerging clinical trials. These guidelines have caused the biological mechanisms that associate T2D and breast cancer to be understudied. Recently, it has been illustrated that the breast tumor microenvironment (TME) composition and architecture, specifically the surrounding cellular and extracellular structures, dictate tumor progression and are directly relevant for clinical outcomes. In addition to the epithelial cancer cell fraction, the breast TME is predominantly made up of cancer-associated fibroblasts, adipocytes, and is often infiltrated by immune cells. During T2D, signal transduction among these cell types is aberrant, resulting in a dysfunctional breast TME that communicates with nearby cancer cells to promote oncogenic processes, cancer stem-like cell formation, pro-metastatic behavior and increase the risk of recurrence. As these cells are non-malignant, despite their signaling abnormalities, data concerning their function is never captured in DNA mutational databases, thus we have limited insight into mechanism from publicly available datasets. We suggest that abnormal adipocyte and immune cell exhaustion within the breast TME in patients with obesity and metabolic disease may elicit greater transcriptional plasticity and cellular heterogeneity within the expanding population of malignant epithelial cells, compared to the breast TME of a non-obese, metabolically normal patient. These challenges are particularly relevant to cancer disparities settings where the fraction of patients seen within the breast medical oncology practice also present with co-morbid obesity and metabolic disease. Within this review, we characterize the changes to the breast TME during T2D and raise urgent molecular, cellular and translational questions that warrant further study, considering the growing prevalence of T2D worldwide.

## Introduction

**Figure 1:**
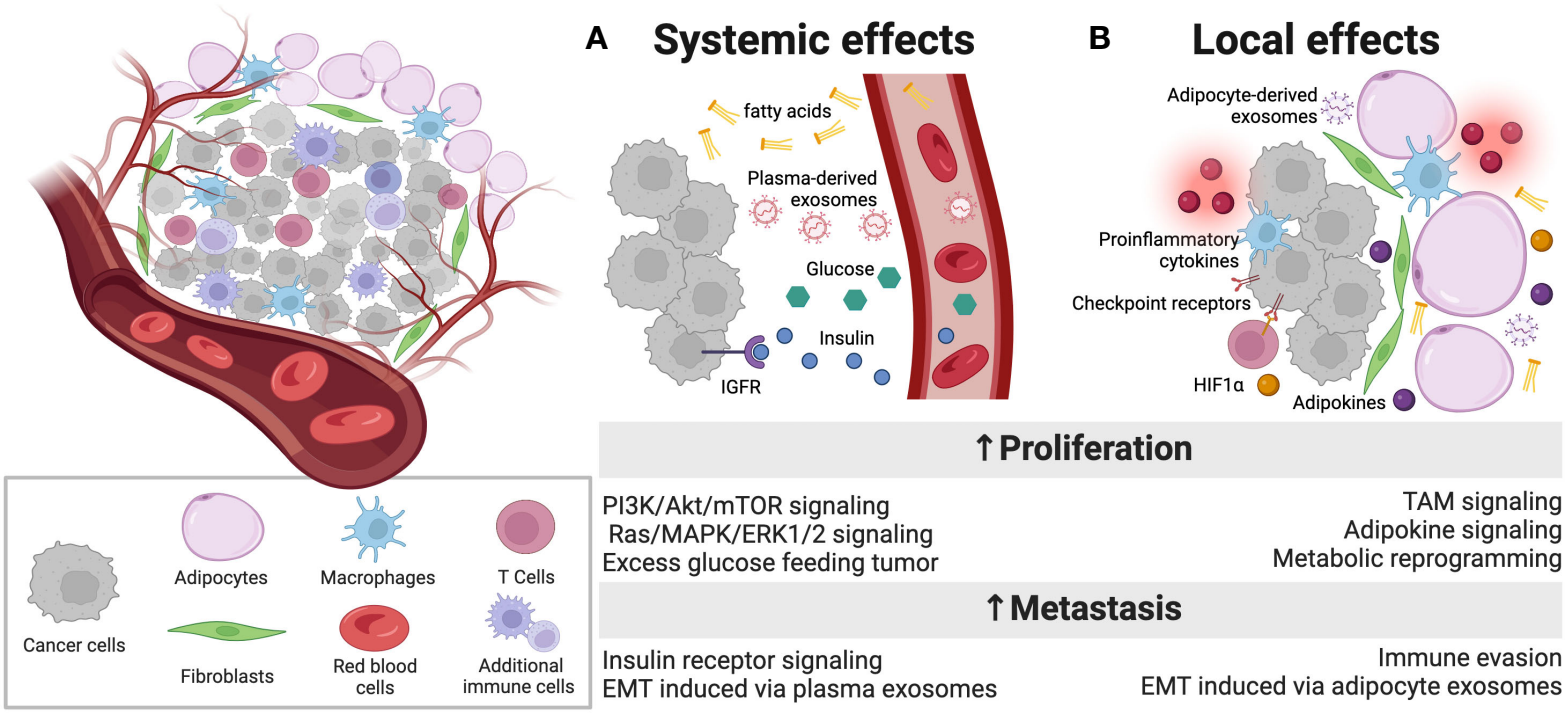
Overview of dysfunction within the diabetic breast TME. A complex array of cell types in the TME engages intercellular communication. **(A)** In addition to well described, systemic factors that are present at abnormally high levels in patients with obesity-driven diabetes, such as free fatty acids, glucose, insulin and IGF-1, which can promote proliferation, plasma exosomes are also altered in diabetes and carry intercellular instructions that promote tumor progression and metastasis. **(B)** Locally, adipocytes in the TME of diabetic patients are inflamed and dysfunctional, releasing proinflammatory cytokines that can alter the function of immune infiltrates, promoting T cell exhaustion through immune checkpoint engagement. These abnormal adipocytes also release adipokines and exosomes that carry payloads capable of reprogramming tumor cells to more aggressive and metastatic phenotypes. Additionally, fibroblasts respond to elevated levels of HIF1α in the local TME to further support the metabolic reprogramming of tumor cells. Figure created with Biorender.com.

Obesity and metabolic disease poses a deepening challenge in the United States, where the burden of Type 2 diabetes (T2D) or pre-diabetes affects over 100 million adults (1–4). Critically, these diseases are implicated in a variety of cancers, both with an obesogenic environment, such as endometrium, colon, and kidney, as well as less common malignancies such as leukemia, multiple myeloma, and non-Hodgkin’s lymphoma (5). Obesity, while a potent risk factor outright, is highly associated with metabolic derangements that increase the incidence and mortality of such cancers (6). However, there are key groups of patients that exhibit counter-intuitive patterns of cancer development: metabolically-healthy obese and metabolically-obese normal weight, which are associated with a reduced and increased cancer prevalence, respectively, compared to metabolically-healthy normal-weight controls (7–10). The link between T2D and breast cancer is of particular significance, with diabetic women not only having a 40% increased risk of developing breast cancer compared to non-diabetic (ND) women, but also a 74% increase in overall mortality (2, 4). This high mortality rate is associated with more advanced stages and aggressive subtypes of breast cancer, such as estrogen receptor negative (ER-) and triple negative breast cancer (TNBC) (11, 12). Despite this significant risk factor, the cellular and molecular mechanisms underlying this comorbidity remain poorly understood and understudied. The combination of these illnesses is particularly challenging due to their heterogeneity and interconnectedness. Recent developments in spatial omics and multiplexed imaging technologies have revealed that cancers have complex spatial organization within their three-dimensional (3D) architectures that dictate a given cell’s spatial neighborhood, interactions, and phenotype to influence overall tumor behavior. The tumor microenvironment (TME), consisting of the cellular and extracellular structures surrounding cancer cells, has been identified to regulate essential tumor survival functions (13, 14). However, standard molecular tools, such as Oncotype, which are used in the clinic to assess personalized risk of recurrence do not account for the profound TME differences seen in T2D patients, with only one measure of mammary adipose inflammation (*CD68*) and no way to account for differing metabolism. Thus, patients with comorbid T2D may receive a dangerously low score that inaccurately estimates their true risk of progression and metastasis. Oncologists therefore urgently need improved diagnostic and therapeutic methods for patients with this comorbidity. Considering that diseased cells immediately adjacent to tumor are not passive structures but instead are active actors in tumor progression, we describe the impact of the TME on this complex yet increasingly common comorbidity (**Figure 1**).

## Systemic effects

Insulin resistance-related metabolic reprogramming, a context-dependent and dynamic process that results from interactions between cancer cells and their local and systemic environments, includes three main aspects, 1) hyperinsulinemia, 2) hyperglycemia, and 3) dyslipidemia (15, 16).

Hyperinsulinemia is often seen in T2D as a result of insulin resistance, in which an impaired tissue response to insulin results in the pancreas increasing insulin levels to compensate and manage blood glucose levels. The significance of insulin signaling towards breast cancer progression has long been noted (17). Primarily, tumor cells widely overexpress insulin receptors (IRs) and insulin-like growth factor receptors (IGF-Rs) (18–21). These receptors can directly bind circulating insulin to activate downstream signaling pathways, such as PI3K/AKT/mTOR and the Ras/MAPK/ERK pathways, to increase mitosis and therefore cancer cell proliferation and invasion (22–29). Subsequent activation of the β-catenin signaling pathway *via* PI3K/AKT has been associated with cancer stemness and chemoresistance (30). Downregulation of IRs on tumor cells has been demonstrated to reduce tumor growth and lung metastasis in xenograft models of athymic mice (31). Moreover, though IR expression is highly expressed in the majority of early stage breast cancers, this expression is not clearly downregulated in the context of hyperinsulinemia (32). Additionally, IGF binding proteins, which limit the activity of IGF-1, are reduced in the presence of high levels of insulin (33, 34). Moreover, hyperinsulinemia can in turn increase IGF-1 expression in the liver and stimulate cell growth (18). Inhibition of IGF-IR has been shown to decrease growth of breast cancer *in vitro* (35, 36).

While hyperinsulinemia is seen as a primary causal factor for cancer, hyperglycemia has also been shown to positively associate with cancer incidence (37). It is well established that tumor cell proliferation needs glucose as an important source of fuel for ATP production as well as synthesis of DNA *via* the pentose-phosphate pathway (18). Further, hyperglycemia can promote epithelial-to-mesenchymal transition (EMT) to induce metabolic reprogramming by upregulating glucose uptake and lactate release (38, 39). As such, multiple large cohort and case-control studies have found that hyperglycemia is positively correlated with the risk of cancer (40–43). Critically, hyperglycemia does not exert a uniform effect on tumor growth in all *in vivo* models. For example, insulin-independent hyperglycemia increases the size of liver tumors and reduces apoptosis in a tumor-prone animal model, whereas in T1D *in vivo* models tumor growth is reduced by insulin (44, 45). However, improved glycemic control with compounds such as metformin has a mixed effect on cancer risk in diabetic patients, indicating that hyperglycemia may be an independent risk factor for cancer (46–51).

Dyslipidemia, characterized by elevated circulating levels of cholesterol, triglycerides, and free fatty acids, is also independently associated with an increased cancer prevalence (20, 52). Both elevated low-density lipoprotein and reduced high-density lipoprotein levels, the main transporters of cholesterol, have been demonstrated to be prognostic factors of breast cancer initiation, progression, and metastasis regardless of metabolic status (53–59). Cholesterol-lowering agents, such as lipophilic statins, have been shown to be protective against breast cancer recurrence and death (59). 27-hydroxycholesterol (27HC) is a primary metabolite of cholesterol, generated upon exposure to cytochrome P450 oxidase sterol 27-hydroxylase A1 (CYP27A1), a key enzyme in regulating cellular cholesterol homeostasis (59–61). 27HC acts as an ER agonist, activating the PI3K/AKT/mTOR and beta-catenin signaling pathways to stimulate cell proliferation and protein synthesis in ER-positive breast cancer (59, 61). Critically, high levels of CYP27A1 expression correlate with high-grade breast tumors, while inhibition of this enzyme reduces tumor growth in hormone-dependent breast cancer (61, 62). Cholesterol, triglycerides, and fatty acids are known to be critical lipid constituents of the cell, composing a majority of the cellular membrane. Highly proliferative cancer cells therefore benefit from the altered lipid metabolism associated with dyslipidemia to provide these essential building blocks (52, 63–66).

## Local effects

In addition to global changes in signaling pathways, breast cancer with comorbid T2D also results in perturbations to the local TME. The breast TME is composed of several cell types that can all experience unique dysfunctions that work to promote tumor proliferation, invasion, and metastasis.

### Adipocytes

Adipocytes are the most prevalent cell type by mass within the breast TME (67). Cancer-associated adipocytes (CAAs) have been demonstrated to promote breast cancer progression (68–70), as they function as an active endocrine tissue to release adipokines (e.g., IL-6, TNFα, leptin, and adiponectin) that can suppress an active immune response (71) and play critical roles in tumor cell proliferation, as well as matrix metalloproteinases that are important for tumor invasiveness (72). T2D and obesity are major contributing factors of inducing adipocyte abnormalities, which are known to promote cancer cell proliferation, invasion, and resistance to chemotherapy and radiotherapy (6). Breast cancer cells adapt to their unique TME to meet their needs for proliferation and cell survival *via* reprogramming their metabolic pathways (70, 73, 74). Accordingly, it has been reported that fatty acid oxidation and pathways required for formation of cell membranes and storage are upregulated in breast cancer (75, 76). In accordance with the need for fuel in proliferation, breast cancer cells develop ways to utilize FAs, such as *de novo* fatty acid synthesis (77). In human epidermal growth factor receptor 2 positive (HER2+) breast cancers, key enzymes involved in this process, fatty acid synthase and acetyl-CoA-carboxylase-a, are upregulated *via* the PI3K/Akt/mTOR pathway (65, 77). Breast cancer cells obtain fuel from its TME through an increased uptake of FAs from CAAs, which requires lipoprotein lipase and fatty acid binding protein 5 and 7, which are all overexpressed in TNBC (53, 77, 78). Despite the significance of CAA-mediated lipid transfer towards breast cancer progression, experiments utilizing primary CAAs from patients with comorbid T2D, who would be experiencing dyslipidemia and having these pathways further perturbed, is lacking. Muller and others have demonstrated that TNBC and ER+ breast tumor cells and nearby CAAs, particularly at the tumor’s invasive front, are likely to engage in crosstalk in a spatially organized manner that elicits tumor EMT and cancer stem-like cell (CSC) formation (79–81). Hursting and colleagues have demonstrated that obesity promotes these two pathways, thus suggesting a causal link between obesity and the associated metabolic derangement with TNBC development (82). However, the contribution of other key adipokines and cytokines in this obesity-associated CSC/EMT circuit must be further examined, as preliminary studies indicate that the leptin-adiponectin ratio imbalances do not fully account for all of the observed effects of diet-induced obesity on TNBC (83).

During an investigation into the role of CAAs in breast cancer progression, our group has recently implicated crosstalk from adipocyte-derived exosomes in driving EMT and cancer aggressiveness. Interest in exosomes, long ignored and thought to be merely cellular disposal systems, has recently been growing. Adipocyte-derived exosomes contain a significant payload of microRNAs that, when applied in a co-culture system with breast cancer cell lines, upregulate genes involved in CSC formation and invasion. Provocatively, fold-changes in these gene expression patterns were greater if the adipocytes had first been rendered insulin resistant or were isolated from patients with T2D (67). This finding supports the idea that the TME is likely more dangerous, leading to increased incidence and metastasis, when the patient has comorbid T2D, which is consistent with observations made in the Black Women’s Health Study (84). A similar phenomenon has been observed in mouse models of diet-induced obesity using breast cancer cell lines in E0771, where obesity causes insulin resistance and metabolic abnormalities in adipocytes that promote expansion of metastasis (85).

### Fibroblasts

Fibroblasts, also known as cancer-associated fibroblasts (CAFs), are the most abundant cell type in the breast TME (86). These cells are derived from resident fibroblasts and a diverse population of mesenchymal cells upon exposure to proinflammatory cytokines such as TNFα and IL-1β (86, 87). Such cytokines are prevalent in the diabetic TME as they are secreted by diseased adipocytes, though little is known about how this may influence the development of CAF outgrowth (88). Provocatively, recent work from Zhu and colleagues report that adipocytes can de-differentiate into fibroblast-like precursor cells during breast tumor progression, with the ability to transform into functional pro-tumorigenic stromal cells such as myofibroblast- or macrophage-like cells (89). Though this phenomenon has not been identified in the context of T2D, the impact of metabolically impaired adipocytes on this mesenchymal transition and subsequent breast cancer progression demands further study. Once differentiated, CAFs exist as highly heterogeneous components of the TME that secrete a variety of soluble factors, such as chemokines and growth factors, that promote both tumor initiation, progression, and invasion (87, 90–92). A key pro-tumorigenic program of CAFs is their function as metabolic support for proliferating tumor cells (93, 94). Their catabolic phenotypes are induced by high levels of reactive oxygen species during oxidative stress upon hypoxia-inducible factor 1α (HIF1α) and nuclear factor κ B (NFκB) signaling within the TME (95). This metabolic shift towards lactate and pyruvate production works to fuel biosynthetic pathways of cancer cells, which can then rely on CAFs to provide a nutrient-rich TME (96). Critically, increased levels of lactate within the TME is known to acidify the area, thus inhibiting effector T cell function and potentially contributing to the failure of anti-tumor therapy in these patients (97–100). HIF1α stability and function are dysregulated by hyperglycemia, and disruption of this aberrant signaling can improve insulin sensitivity (101–104). Within the diabetic breast TME, higher levels of HIF1α and subsequent oxidative stress contribute to hypoxia (101, 103, 105). Hypoxia is a driving force of tumor progression as it stimulates vascularization *(VEGF, ANG1, ANG2, MMPs, LOX, CAIX, CXCR4)*, upregulates EMT/CSC signatures *(SNAI1, SNAI2, TWIST1, SOX9, SOX2, OCT4, NANOG)*, and contributes to drug resistance (106–109). Despite the importance of metabolic reprogramming in cancer biology, the role of CAFs in the diabetic TME have yet to be fully investigated.

### Macrophages

Macrophages form a critical and diverse component of the breast TME (86, 87). They can exist as tissue resident cells or differentiate from circulating monocytes that are recruited to the tumor site *via* chemokines secreted by cancer and stromal cells (86, 87, 110). Once in the breast TME, macrophages can have both proinflammatory (M1-like) and anti-inflammatory (M2-like) functions. During normal immunological responses, most macrophages are leaning towards the M1-like phenotype and engage in responses to pathogens (111). The M2-like phenotype, however, is associated with T helper 2-type cytokines and is typically associated with wound healing and tissue remodeling (111). Most tumor-associated macrophages (TAMs) are leaning towards the M2-like phenotype as this class promotes cell proliferation and breast cancer progression *via* anti-inflammatory signaling pathways (*IL10, CCL2, CCL17, CCL22, TGFB*) (112). TAMs have been reported to support invasion and metastasis by secreting EGF1 and TNFα, which promote EMT and enhance the stemness and angiogenesis of cancers (113). Many studies have linked high TAM levels to a worse prognosis, suggesting that TAM depletion or reprogramming may serve as a key therapeutic target (114, 115). The common anti-diabetic drug metformin has been shown to modulate macrophage polarization, specifically by decreasing the percent of M2-like and increasing the percent of M1-like macrophages, through AMP-activated protein kinase (AMPK)-NFκB signaling (116). This suggests that metformin could be therapeutically advantageous in facilitating macrophage reprogramming.

Within the diabetic and obese TME, macrophages are known to play a critical role in local adipocyte inflammation. M1-like macrophages accumulate within adipose tissue and produce factors that deregulate adipocyte signaling processes, increase production of reactive oxygen species, and potentiate insulin resistance (110, 117–119). They can form a characteristic crown-like structure around the hypertrophied and dying adipocytes (110, 118, 119). This mammary adipose tissue inflammation is thought to contribute to the link between metabolic derangements and worse breast cancer prognosis (110, 117, 120–122). Critically, in patients with breast cancer and comorbid obesity, the presence of crown-like structure accumulation is associated with more aggressive, high-grade tumors (110, 114, 117). Despite these demonstrated functions, additional studies are needed to understand the potential clinical utility of crown-like structure accumulation as a biomarker of breast cancer risk or prognosis.

### T cells

Another major component of the breast TME are tumor-infiltrating lymphocytes (TILs) (123–125). Importantly, although inflammation regulation in T2D has centered around macrophages, recent evidence suggests that T cells are vital for metabolic inflammation and insulin resistance associated with the disease (123, 126–131). The majority of TILs are T cells, which can be further subdivided into CD4+ helper and CD8+ cytotoxic T cells. The ratio between these two TIL populations is a critical prognostic indicator of breast cancer progression, with infiltration of CD8+ T cells associated with longer survival (124, 132–135). Miya and colleagues demonstrated that diabetic patients exhibit a decreased proportion of peripheral CD8+ T cells after glucose loading compared to nondiabetic control subjects (136). However, it is unclear how this finding may extend to the local TME of breast cancer patients with comorbid T2D. The effect of these cell populations is regulated by a balance between co-stimulatory and co-inhibitory signals at immune checkpoints. These regulatory pathways, involving molecules such as programmed death-1 (PD-1), typically work to inhibit T-cell function in order to prevent inappropriate immune reactions (137–139). However, these pathways are known to be hijacked by tumor cells to evade immune detection and clearance (137–139). Tumors are able to upregulate the expression of cognate ligands, such as programmed death-ligand 1 (PD-L1), on their cell surface, which reprogram local TILs towards an inhibitory state known as immune exhaustion (137–139). Immune exhaustion is a unique differentiation state for T cells, in which they become metabolically impaired and lose their effector functions to create an immunosuppressive TME (137–139). Both PD-1+ TILs and PD-L1+ tumors are associated with a worse prognosis for breast cancer patients (123, 124). Critically, circulating T cells in diabetic patients are known to have high surface expression of PD-1 (137, 140, 141). Immunotherapeutic strategies that aim to reverse this exhausted microenvironment have been gaining traction recently. Studies have shown that treatment with the anti-PD-1 agent pembrolizumab, in combination with chemotherapy as neoadjuvant therapy, resulted in a significant reduction in the risk of disease progression for patients with high-risk early-stage TNBC (142–145). However, little research has been done into whether breast cancer patients with comorbid T2D benefit from these same effects. Deepening our understanding of TIL biology and the role of exhaustion in T2D and breast cancer may be key in unraveling underlying disparities.

### Spatial organization

Recent developments in spatial transcriptomic technologies have revealed that cancers have complex spatial organization within their three-dimensional architectures that dictate a given cell’s spatial neighborhood, interactions, and phenotype to influence overall tumor behavior (13, 14). Of note, immune responses, such as immunoregulatory pathways, are highly spatially organized processes within the breast TME (13, 146). Specifically, regulatory T and exhausted T cells co-occur in space with highly proliferative tumor cells, linking this spatially suppressed TME to poor patient outcomes (147). In addition, the adipocytes immediately adjacent to a breast tumor are known to be active actors in tumor progression, with investigators beginning to probe secretory and spatial relationships among breast adipocytes in invasive cancers with histological evidence of crosstalk (79–81). The spatial organization and transcriptional relationships among breast cancer cells and nearby adipocytes, particularly at the tumor invasive front, are likely to engage in exosome crosstalk that elicits tumor EMT and CSC formation. Taken together, the TME is likely more dangerous when a breast cancer patient has comorbid T2D. Deepening our understanding of how this increasingly common comorbidity may impact spatial heterogeneity, architecture, and signaling will be critical in improving therapeutic outcomes for these patients.

## Treatment opportunities

The first-line medication for treatment of T2D is metformin, a biguanide drug that lowers glucose production by the liver through inhibiting the mitochondrial respiratory chain, activating AMPK, lowering cAMP, and reducing the expression of gluconeogenic enzymes, thus enhancing insulin sensitivity (148). Even though metformin has been used for more than 60 years in the clinic, several studies have demonstrated new indications and mechanisms of action for the drug as an anti-tumor agent (149). Given that metformin activates AMPK, it thereby inhibits mTOR pathways and decreases circulating insulin levels, with hyperinsulinemia being tied to worse breast cancer prognosis. It also inhibits the proliferation and invasion of cancer cells, which could limit metastatic spread (150–155). The Adjuvant Lapatinib and/or Trastuzumab Treatment Optimization trial tested metformin use in HER2+ breast cancer, showing an improvement in prognosis (156, 157). Other clinical studies have correlated metformin use with improved breast cancer-specific survival in HER2+ breast cancer, though not in TNBC (158). Goodwin and colleagues recently reported (159) a lack of survival benefit of metformin for either ER/PR+ or ER/PR- breast cancer patients. We consider that these results are skewed due to the exclusion of T2D patients and lack of stratification by patient BMI as improper exclusion criteria by considering all patients metabolically healthy, therefore the results are unsurprising. However, recent work has demonstrated that activation of AMPK upregulates the expression of EMT and stemness genes (*NANOG, SOX2, BMI1*), through the transcriptional upregulation of *TWIST1* (160). This AMPK-driven stemness has been shown to play an important role in breast cancer drug resistance, thus complicating the effect metformin may have on breast cancer. This unexpected, potentially dangerous pathway of AMPK activation by metformin, which appears to increase survival of circulating metastatic breast cancer cells, demands further study.

Metformin has also been shown to have a beneficial effect in the regulation of T cell functions, providing a potential therapeutic for immune exhaustion *via TSC1*/mTOR (149). Many previous studies have linked the anticancer effects of metformin to the differentiation of T cells (149, 161). For instance, the use of metformin has been reported to increase the expression of CD8 and CD69 while decreasing PD-1 in TILs, thus increasing the number of CD8+ T cells while protecting them from apoptosis and exhaustion (161). Cytokines seem to be key in this process, as metformin upregulates the secretion of interferon γ, IL-2, and TNFα *via* AMPK (162, 163). Since the recent emergence of microRNAs as crucial regulators of T cell differentiation, there are reports showing metformin increasing miR-7 expression in an AMPK-dependent pathway and inhibiting the action of miR-107, which is linked to insulin sensitivity and the expression of PD-1 (161). Several ongoing clinical trials are examining whether metformin has benefit in the context of immune checkpoint blockade in treatment of solid tumor, such as NCT03048500 for non-small cell lung cancer, and NCT03800602 for refractory, microsatellite-stable colorectal cancer.

Overall, though most current studies that examine metformin’s use in breast cancer have reported a mixed picture on its efficacy, they demonstrate a potential therapeutic benefit of metformin in patients with breast cancer and comorbid T2D. It is clear that metformin holds considerable promise with regard to a potential antitumor agent.

## Conclusions and future directions

Over 100 million Americans with T2D or pre-diabetes are predisposed to more aggressive tumors, but the mechanistic basis for this differential risk remains unclear and understudied (1). Herein, we have outlined the current state of knowledge on the relevant cell types and mechanisms underlying this comorbidity.

However, many questions remain on how cell diversity within the diabetic breast TME may impact tumorigenesis, proliferation, or even drug resistance in these patients. For example, it is unclear how related obesity, *via* an increase in dysfunctional adipocytes, may change the heterogeneity of the TME and how any subsequent changes may support a diversity of transcriptional states key in pro- and anti-tumorigenic processes. Furthermore, understanding the interplay between the TME and T2D is further complicated by the need to simultaneously understand how intercellular crosstalk is organized at the tissue level and how multiple regulatory layers of cellular identity might play distinct roles. As previously stated, these layers can encompass alterations in cellular composition, tissue architecture, RNA expression, cytokine expression, lipid content, metabolomics, and other types of molecular analytes that work in concert to create the underlying disease phenotypes. Nevertheless, with the advent of single cell RNA sequencing and, specifically spatial omics and multiplex imaging technologies, the field of oncology is entering a new era of technological innovation where the necessary multimodal spatial datasets can be created that will aid in providing the necessary systems-level understanding of these complex and multifaceted disease phenotypes and interactions. Thus, these approaches offer novel methods in studying breast cancer as well as other cancer types differentially impacted by T2D. Further investigating the effects of the metabolically disturbed TME cell types on intercellular communication and cancer pathogenesis will be critical in identifying biomarkers and novel therapeutic targets for patients with breast cancer and comorbid T2D. These patients have been excluded and clinical trial design should be adapted among cancer disparities consortia to include them in well-defined groups with sufficient statistical power. We propose that investigating the mechanisms of intercellular crosstalk with tumor cells in a T2D setting is crucial to fully understand how this comorbidity might be working, integrating metabolic and immune exhaustion signatures like tumor progression, metastasis, and immune checkpoint. Utilizing this holistic approach will be crucial in revealing novel insights into tumor progression and metastasis in T2D patients and adopting multiple levels and perspectives of metabolism and intercellular communication within the TME.

## Author contributions

Conceptualization, CE, RD, and GD. Writing, CE, PL, and YQ. Revision and editing, CE, RD, and GD. All authors contributed to the article and approved the submitted version.

## Funding

National Cancer Institute (U01CA182898, R01CA222170; GV Denis).

## Conflict of interest

The authors declare that the research was conducted in the absence of any commercial or financial relationships that could be construed as a potential conflict of interest.

## Publisher’s note

All claims expressed in this article are solely those of the authors and do not necessarily represent those of their affiliated organizations, or those of the publisher, the editors and the reviewers. Any product that may be evaluated in this article, or claim that may be made by its manufacturer, is not guaranteed or endorsed by the publisher.

## Abbreviations

27HC, 27-hydroxycholesterol; AMPK, AMP-activated protein kinase; CAA, cancer-associated adipocyte; CAF, cancer-associated fibroblast; CSC, cancer stem-like cell; CYP27A1, cytochrome P450 oxidase sterol 27-hydroxylase A1; EMT, epithelial-to-mesenchymal transition; ER, estrogen receptor; HER2, human epidermal growth factor receptor 2; HIF1α, hypoxia-inducible factor 1α; IGF-1R, insulin like growth factor 1 receptor; IR, insulin receptor; NFκB, nuclear factor κ B; PD-1, programmed death-1; PD-L1, programmed death-ligand 1; PI3K/Akt/mTOR, phosphoinositide 3 kinase/Akt/mammalian (or mechanistic) target of rapamycin; Ras/MAPK/ERK1/2, Ras/mitogen-activated protein kinase/extracellular signal-related kinase 1/2; T2D, type 2 diabetes; TAM, tumor associated macrophage; TIL, tumor infiltrating lymphocyte; TME, tumor microenvironment; TNBC, triple negative breast cancer.
